# Gastric cancer in Abidjan: care strategies and survival in a resource-limited setting

**DOI:** 10.3389/fonc.2026.1737304

**Published:** 2026-03-18

**Authors:** Kouamé Konan Yvon Kouassi, Yénahaban Lazare Touré, Bitti Addé Odo, Pétiori Gningayou Laurence Touré, N’Guessan Manlan Prosper Mébiala, Fleur Audrey Sességnon, Akissi Marie Barbara Yvonne Nogbou, Mohamed Kassir Agnidé Madiou, Ibrahima Alhassane Cissé, Moctar Touré, Innocent Adoubi

**Affiliations:** 1 Medical Oncology Department, Treichville University Hospital Center, Abidjan, Côte d’Ivoire; 2 Félix Houphouët-Boigny University, Faculty of Medicine, Abidjan, Côte d’Ivoire; 3 Medical Oncology Department, Bouaké University Hospital Center, Bouaké, Côte d’Ivoire; 4 Alassane Ouattara University, Faculty of Medicine, Bouaké, Côte d’Ivoire; 5 National Cancer Control Program, Abidjan, Côte d’Ivoire

**Keywords:** Abidjan, Côte d’Ivoire, gastric cancer, management, survival

## Abstract

**Background:**

In Côte d’Ivoire, the management of gastric cancer relies mainly on chemotherapy and surgery. However, the actual impact of these treatments on patient survival remains poorly documented.

**Objective:**

To evaluate management strategies and clinical outcomes of patients with gastric cancer in Abidjan.

**Methods:**

A 5-year retrospective cohort study was conducted across five hospitals in Abidjan. All patients followed for gastric cancer were included. Clinical, therapeutic, and outcome data were analyzed using SPSS, with a significance threshold set at p < 0.05.

**Results:**

Seventy-seven patients were included (mean age: 56.9 years; male-to-female ratio: 2.8). Consultation was often delayed (>3 months after symptom onset). Metastatic disease accounted for 60.7% of cases. Adenocarcinoma was the predominant histological type (98.7%). A multidisciplinary tumor board (MTB) was held in only 37.7% of cases. Initial treatment was surgical (49.4%), palliative/supportive (26%), or chemotherapy-based (23.4%). Median survival was 15 months. Disease stage at diagnosis and consultation delay significantly influenced survival.

**Conclusion:**

Gastric cancer is managed in Abidjan, but major gaps remain, particularly the lack of systematic MTB discussion and late diagnosis. Broader access to innovative therapies and the implementation of early detection strategies are urgently needed to improve outcomes.

## Introduction

Gastric cancer is a malignant tumor of the stomach, distinct from gastroesophageal junction cancers by its anatomical location and histological characteristics (1). It represents a major public health challenge, with nearly one million new cases and more than 660,000 deaths worldwide in 2022, ranking as the fourth most common cancer and the fifth leading cause of cancer-related mortality (2). In West Africa, particularly in Nigeria, gastric cancer accounts for 1.6–4.5% of diagnosed cancers, predominantly affecting men (3). In Côte d’Ivoire, it is the fifth most frequent cancer in terms of both incidence and mortality (2).

Major risk factors include *Helicobacter pylori* infection, tobacco use, nitrate-rich diets, and certain genetic predispositions (4, 5). In resource-limited countries, diagnosis is often made at an advanced stage, which restricts therapeutic options and reduces survival prospects (2, 6). The early screening is particularly critical in patients presenting with persistent epigastric pain, as this common symptom may represent the first clinical sign of gastric cancer. Management generally relies on a multidisciplinary approach including surgery, chemotherapy, radiotherapy, targeted therapies, and supportive care by integrating an early nutritional assessment, an essential strategy in gastric cancer (7, 8). Nevertheless, the 5-year survival rate remains below 30% in most countries, except in regions with established screening programs such as Japan and South Korea (9).

In Côte d’Ivoire, limited resources and the absence of an organized screening program make patient management challenging and often incomplete (10, 11). To date, few local studies have assessed the actual impact of available treatments on survival outcomes. This study aims to fill this gap by describing the clinical, therapeutic, and survival characteristics of gastric cancer in national referral centers.

## Materials and methods

### Study design and setting

We conducted a retrospective analytical cohort study in the main referral hospitals involved in gastric cancer management in Abidjan, Côte d’Ivoire. These centers represent the country’s leading institutions for gastric cancer care. The study period extended from January 1, 2018, to December 31, 2022, corresponding to the patient recruitment period.

### Study population and inclusion criteria

Patients aged 18 years or older with histologically confirmed gastric cancer who initiated treatment (surgery, chemotherapy, or supportive care) with a minimum medical follow-up of 3 months after treatment initiation between January 1, 2018, and December 31, 2022, were eligible.

The 77 included patients do not represent all incident gastric cancer cases during the study period, but only those with confirmed diagnosis, treatment initiation, and available follow-up data. A substantial number of patients were lost prior to histological confirmation or treatment initiation due to delayed consultation, financial constraints, or referral difficulties.

### Variables studied

The collected variables were grouped into four categories:

- Sociodemographic data: age, sex, occupation, education level, marital status, residence, risk factors, comorbidities.
- Clinical data: circumstances of diagnosis, diagnostic delay, WHO performance status, histological type, disease stage.
- Therapeutic data: treatment initiation date, type and modality of treatment, tumor response according to RECIST criteria, treatment adherence.
- Outcome data: date of last follow-up, vital status, survival duration.

### Therapeutic decision-making

Treatment orientation toward surgery, chemotherapy, combined treatment, or supportive care was based on a combination of tumor stage (TNM classification), World Health Organization (WHO) performance status, tumor resectability, nutritional status, availability of multidisciplinary tumor board (MTB) discussion, and socioeconomic constraints.

### Data collection procedure

Patients were identified through both paper-based and electronic databases of the participating centers. A standardized electronic case report form was used to extract relevant information from medical records.

### Endpoints

The primary endpoint was progression-free survival (PFS), defined as the interval between treatment initiation and the date of clinical/radiological progression or death, according to RECIST v1.1. Overall survival (OS) was defined as the time from treatment initiation to death from any cause.

### Handling of missing data

Variables with more than 10% missing data were excluded from multivariate analyses in order to limit statistical bias. This methodological choice may have resulted in the loss of clinically relevant information and was taken into account when interpreting the results.

### Statistical analysis

Data were analyzed using IBM SPSS Statistics.

- Qualitative variables were expressed as frequencies and percentages. The Chi-square test was used to compare proportions, with statistical significance set at p < 0.05.
- Quantitative variables were described by mean and standard deviation.
- Survival curves were estimated using the Kaplan-Meier method.
- Comparisons of survival curves were performed using the log-rank test.
- Factors associated with PFS were assessed in two steps:
  - Univariate analysis using the Cox proportional hazards model.
  - Multivariate analysis including significant or clinically relevant variables, with automated forward selection (stepwise method) based on the Akaike Information Criterion (AIC).

Statistical significance was set at 5%.

### Ethical considerations

This study was conducted in accordance with ethical principles. Data were anonymized to ensure confidentiality and patient privacy.

## Results

### Clinical and pathological characteristics

A total of 77 patients were included. The mean age was 56.9 ± 12.2 years (range: 25–87 years). Males predominated (67.5%; sex ratio: 2.8). The most represented occupational category was manual workers (20.8%), while secondary education level was most frequent (40.2%).

A history of peptic ulcer disease was found in 41.5% of patients. Adenocarcinoma was the predominant histological type (98.7%). None of the patients underwent testing for HER2 expression, PD-L1, or MSI-H status. At diagnosis, the majority (60.7%) already had metastatic disease (**Table 1**).

**Table 1:** Clinical and demographic characteristics of patients with gastric cancer.

Variables		Number (n = 77)	Percentage (%)
Sex			
	Male	52	67.5%
	Female	25	32.5
Age (years)			
	[25-35]	4	5.2
	[36-45]	14	18.2
	[46-55]	18	23.4
	[56-65]	21	27.3
	[66-75]	18	23.4
	[76-87]	6	7.8
Education level			
	No formal education	18	23.4
	Primary	25	32.5
	Secondary	31	40.2
	Higher education	3	3.9
Comorbidities			
	Peptic ulcer disease	32	41.5
	Hypertension	7	9.1
	Diabetes mellitus	2	2.6
	Family history of cancer	1	1.3
	Other comorbidities	4	5.2
Lifestyle factors			
	NSAID use	20	26
	Smoking	14	18.2
	Alcohol consumption	3	3.9
Histopathological type			
	Adénocarcinoma	76	98.7
	Lymphoma	1	1.3

### Therapeutic aspects and outcomes

Only 37.7% of cases were discussed at a multidisciplinary tumor board (MTB). Patients were classified into three main therapeutic groups: surgery alone, including curative or palliative procedures (23 patients, 29.9%); chemotherapy alone, mainly administered with palliative intent (18 patients, 23.4%); and combined treatment associating surgery and chemotherapy in neoadjuvant or adjuvant settings when applicable (20 patients, 26.0%). No patient received targeted therapy or immunotherapy (**Table 2**).

**Table 2:** Therapeutic management of patients.

Variables		Number	Percentage (%)
Case discussed at the MTB (n = 77)			
	No	48	62.3
	Yes	29	37.7
Surgery (n = 77)			
	Yes	41	53.2
	No	36	47.8
Type of surgery (n = 41)			
	Primary surgery	23	46.1
	Palliative surgery	18	43.9
Surgical procedure (n = 41)			
	Partial gastrectomy	23	56.1
	Total gastrectomy	13	31.7
	Exploratory laparotomy	1	2.4
	Palliative gastrectomy	2	4.9
	Jejunostomy	2	4.9
Chemotherapy (n=77)			
	Yes	38	49.4
	No	39	49.6
Type of chemotherapy (n = 38)			
	Palliative	20	52.6
	Adjuvant	12	31.6
	Néoadjuvant	6	15.8
Chemotherapy regimens (n = 38)			
	FOLFOX 4	25	65.8
	FLOT	5	13.2
	ECX	3	7.9
	XELOX	3	7.9
	ECF	1	2.6
	LV5FU2	1	2.6
Radiotherapy (n = 2)			
	alone	1	50.0
	CRT	1	50.0
**Therapeutic strategy**	Surgery alone (curative or palliative)	23	29.9
	Chemotherapy alone (mainly palliative)	18	23.4
	Combined treatment (surgery + chemotherapy, neoadjuvant or adjuvant)	20	26
Other treatments (n = 20)			
	Targeted therapy	0	0
	Immunotherapy	0	0
	Supportive care	20	100

MTB, Multidisciplinary Tumor Board; CRT, Concurrent chemoradiotherapy.

**Figure 1:**
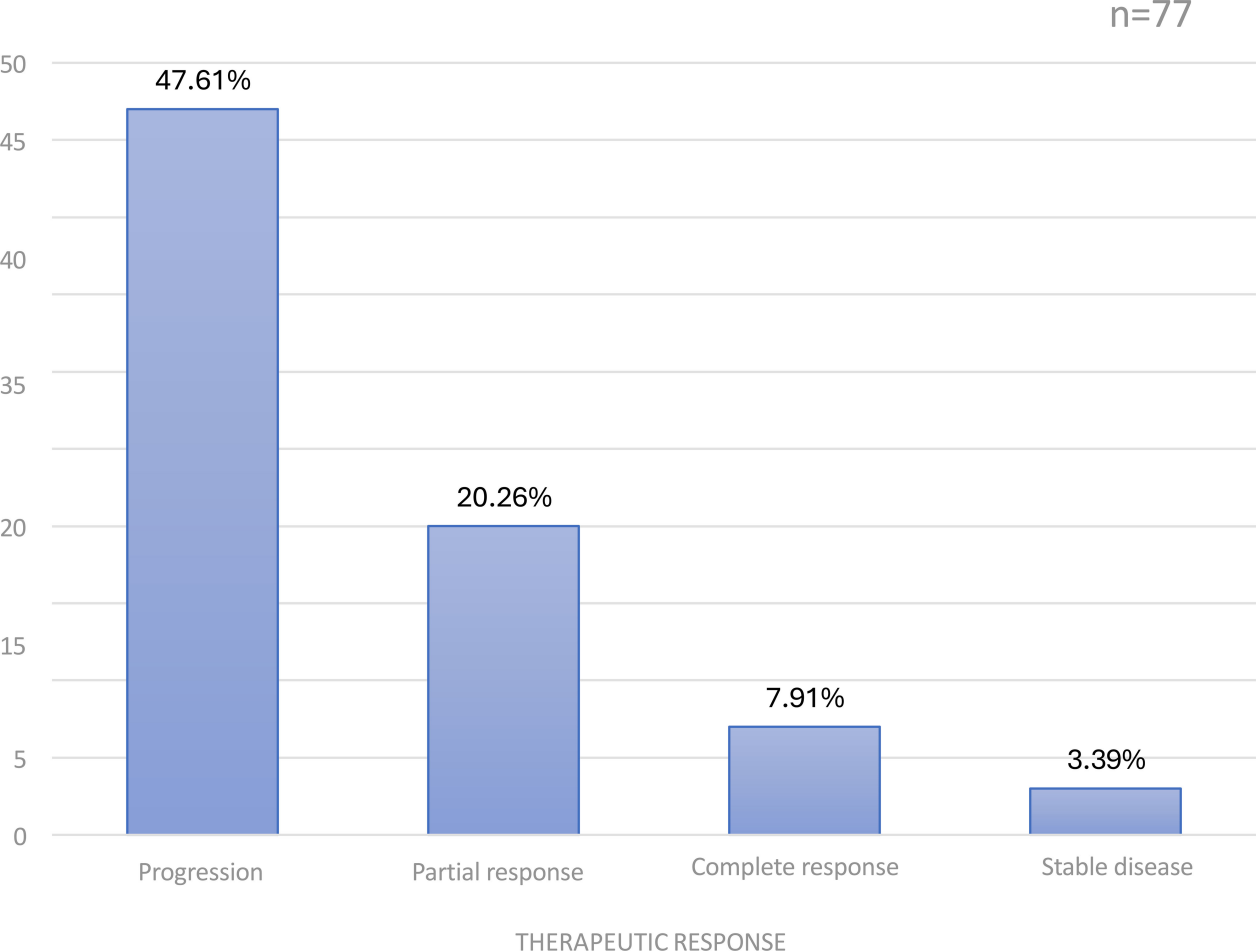
Distribution of patients according to therapeutic response.

Initial treatment consisted of surgery in 49.3% of cases, chemotherapy in 23.4%, and radiotherapy in 1.3%. Partial gastrectomy was the most common surgical procedure (56.1%). Among patients who received chemotherapy, 52.6% received palliative treatment, 31.6% adjuvant therapy, and 15.8% neoadjuvant therapy. The FOLFOX4 regimen was used in 65.8% of cases. Disease progression occurred in 61% of patients, while complete response was observed in 9.1% (**Figure 1**).

### Mortality and associated factors

The analysis of prognostic factors associated with mortality showed that sex was significantly correlated, with male patients having a higher risk of death compared to females (p = 0.007). Age was not statistically associated with mortality, although patients aged 56–65 years had a higher, but non-significant, odds ratio (p = 0.10) (**Table 3**).

**Table 3:** Correlation between epidemiological, clinical, and therapeutic factors and mortality.

Variables	Alive	Deceased	P-value
Sex			
Male	5	24	0.007
Female	8	10	0.007
Age (years)			
25-35	0	2	NA
36-45	3	6	0.5
46-55	4	9	0.26
56-65	2	8	0.1
66-75	4	4	1
75-87	0	5	NA
Educational level			
No formal education	2	13	0.007
Primary	5	6	1
Secondary	6	13	0.167
Higher education	0	2	NA
**Weight loss (%)**	7	13	0.27
≤10%	3	17	0.27
≤10%	9	14	0.405
**Delay in consultation**	2	15	0.002
<3 months	2	5	0.453
3–6 months	9	17	0.169
>12 months	4	17	0.007
Multidisciplinary Tumor Board (MTB)			
Yes			
No			

OR, Odds Ratio; CI, Confidence Interval.

Regarding educational level, patients without formal education had a significantly higher risk of death compared to educated patients (p = 0.007).

Clinical factors also influenced mortality. A weight loss of more than 10% tended to be associated with increased mortality risk, although this did not reach statistical significance (p = 0.27). A consultation delay of 3–6 months was significantly associated with a poorer outcome (p = 0.002) (**Table 3**).

Participation in a Multidisciplinary Tumor Board (MTB) discussion was associated with a significantly increased risk of death (p = 0.007).

Finally, therapeutic modalities showed that chemotherapy and surgery were not significantly associated with mortality. Patients who received only supportive care all died during the study period (**Table 3**).

In the multivariate logistic regression analysis, several factors were evaluated for their association with mortality. Consultation delay longer than six months was significantly associated with increased risk of death (p = 0.020). Tumor stage was also a significant predictor: patients with stage IA–IIA disease (p = 0.030) and those with stage IIB–IIIB disease (p = 0.030) had a significantly higher risk of mortality compared with the reference category (**Table 4**).

**Table 4:** Multivariate analysis of factors associated with mortality.

Variable	OR	95% CI	P-value
Female sex	0.7	[0.4 - 0.8]	0.160
Male sex	8.86	[0.42 - 186.4]	0.160
Consultation delay ≤ 6 months	1.01	[0.01 - 84.81]	0.998
Consultation delay > 6 months	0.01	[0.022 - 0.52]	0.02
Weight loss <10%	0.29	[0.057 - ∞]	1
Weight loss 1-10%	0.75	[0.02 - ∞]	1
Multidisciplinary Tumor Board	0.23	[0.5 - 178]	0.384
Stage IA-IIA	0.02	[0.002 - 0.67]	0.030
Stage IIB-IIIB	0.02	[0.07 - 0.68]	0.030
Stage IV	0.23	[0.01 - 4.43]	0.332

Sex, weight loss, and discussion at the Multidisciplinary Tumor Board (MTB) did not show a statistically significant association with mortality in the adjusted model.

### Survival

**Figure 2:**
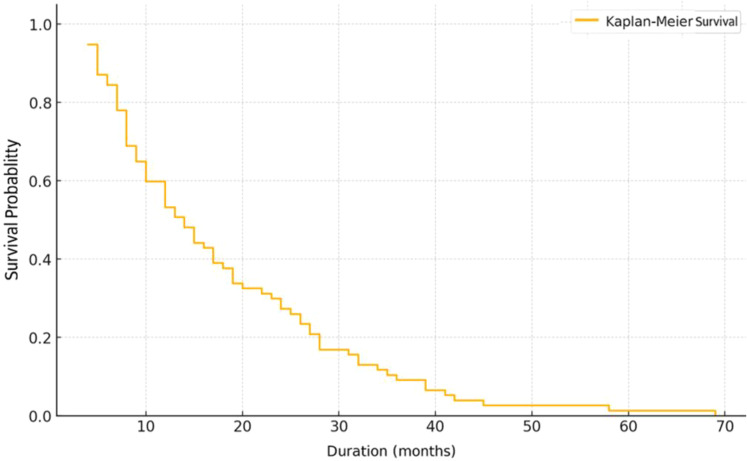
Kaplan Meier overall survival curve.

The Kaplan-Meier survival curve demonstrates a gradual decline in survival throughout the follow-up period. The median survival is estimated at approximately 15 months. The 12-month survival rate is around 50%, decreasing to below 20% after 30 months, and approaching zero beyond 60 month (**Figure 2**).

**Figure 3:**
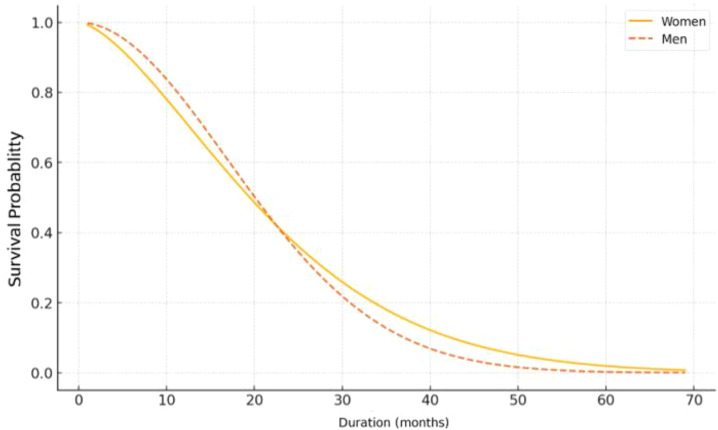
Survival curves by sex.

The Kaplan-Meier survival analysis shows better overall survival in women compared to men. The difference becomes more pronounced after 20–25 months, with women maintaining a higher survival probability. Beyond 40 months, survival in men approaches zero, while a residual probability of survival persists among women (**Figure 3**).

**Figure 4:**
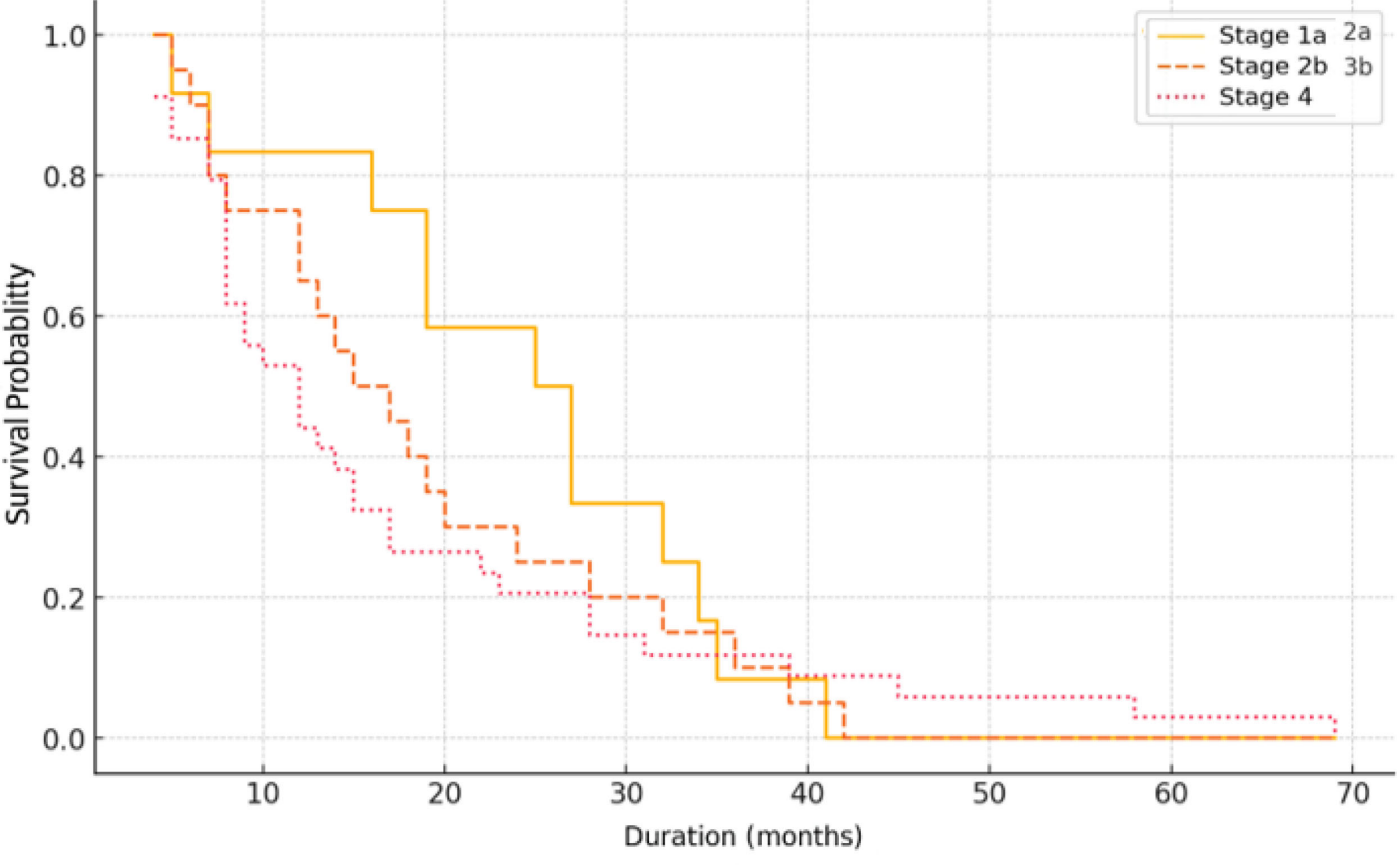
Survival curves by cancer stage.

The Kaplan-Meier survival analysis demonstrates a clear stage-dependent prognosis. Patients diagnosed at early stages (IA–IIA) show the highest survival probability, while those at intermediate stages (IIB–IIIB) have shorter survival. Stage IV patients exhibit the poorest outcomes, with a rapid decline in survival probability (**Figure 4**).

**Figure 5:**
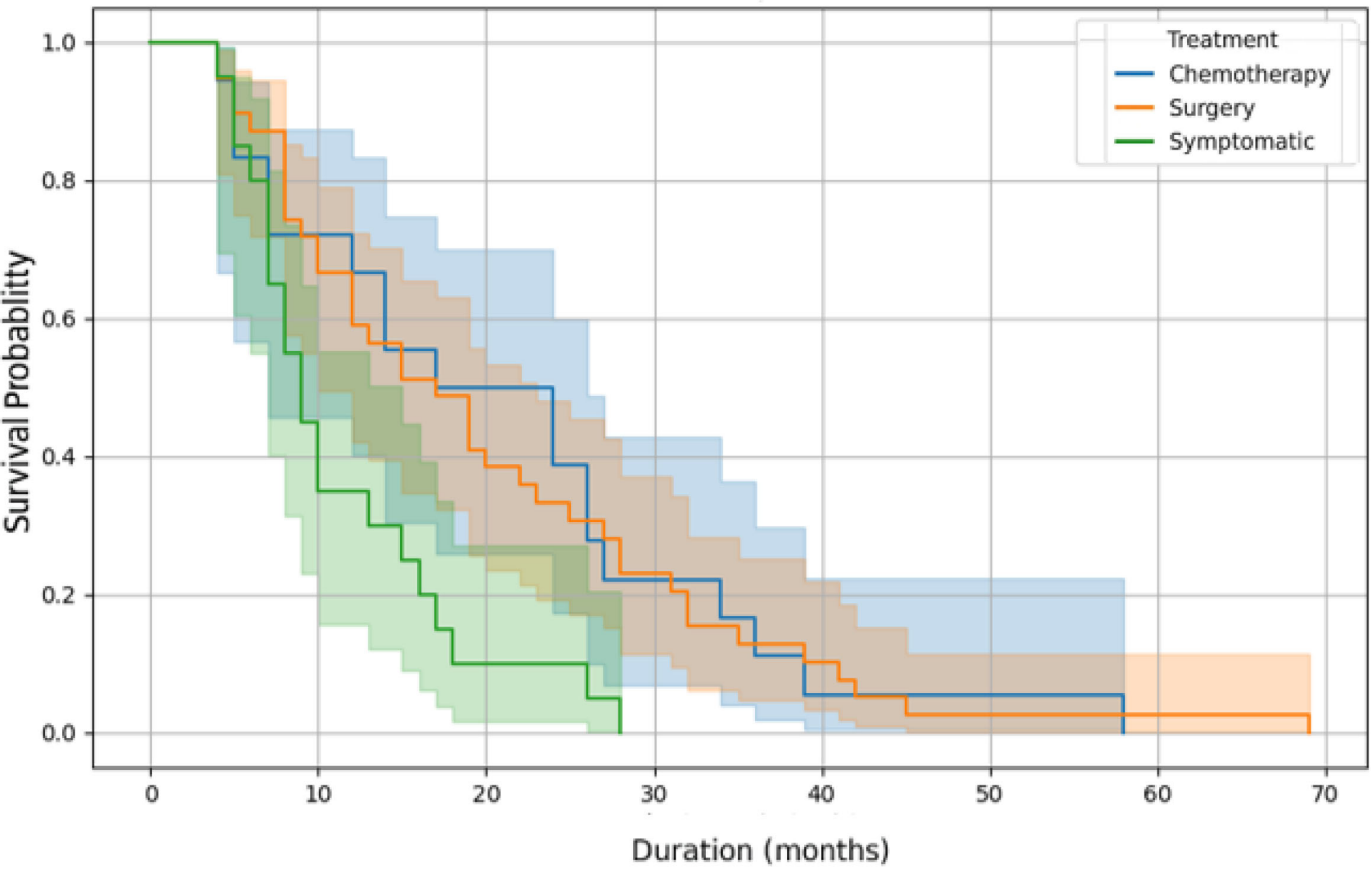
Survival curves according to the initial treatment.

The Kaplan-Meier survival analysis demonstrates differences according to treatment modality. However, no statistically significant differences in overall survival were observed according to treatment modality using log-rank testing. Patients managed with supportive care only showed the shortest survival, but these differences should be interpreted descriptively given the observational nature of the study and the absence of adjustment for disease stage or performance status (**Figure 5**).

These findings indicate that both chemotherapy and surgery are associated with improved survival compared to supportive care alone.

## Discussion

This study provides valuable insights into the management and outcomes of gastric cancer in a resource-limited setting. It highlights suboptimal therapeutic performance, compounded by the lack of access to molecular diagnostic techniques and innovative treatments.

The mean age (56.9 years) and male predominance (sex ratio 2.8) are consistent with studies conducted in Morocco and Mali (12, 13). In the study population, peptic ulcer disease was a frequent antecedent (41.5%), as also observed in other sub-Saharan African countries (14), although prevalence was markedly lower in some Moroccan series (15).

Adenocarcinomas accounted for 98.7% of cases, consistent with global literature (2–14). None of the patients underwent HER2, PD-L1, or MSI-H testing, in contrast with recommendations from societies such as ESMO, INCa, or TNCD. The absence of molecular profiling restricted access to targeted therapy and immunotherapy, likely contributing to the low complete response rate (9.1%) and the high progression rate (61%).

The limited use of Multidisciplinary Tumor Boards (MTB, 37.7%) underscores shortcomings in interdisciplinary coordination. Partial gastrectomy (56.1%) was the most frequently performed procedure, consistent with sub-Saharan data (16), but differs substantially from Japanese practice, where early cancer detection allows curative interventions in most cases (17, 18). The structuring of the care pathway for patients treated with curative intent is organized around the following steps: diagnosis, neoadjuvant chemotherapy, surgery, and adjuvant chemotherapy. The predominant use of the FOLFOX4 regimen (65.8%) aligns with recommended options (5-FU plus platinum), although perioperative FLOT is preferred in curative settings whenever feasible (5, 19, 20).

The overall median survival of 15 months and the 5-year survival rate below 1% reflect a very poor prognosis, consistent with estimates reported in some resource-limited settings (21, 22). By contrast, countries with early detection programs such as Japan and South Korea report 5-year survival rates ranging from 60% to 68.9% (14, 22).

Delayed consultation and advanced disease stage were independently associated with reduced survival, consistent with findings from other studies (5). Advanced age, poor ECOG performance status, and lack of MTB discussion or targeted therapy are also known to influence outcomes, although these could not be assessed in our cohort due to the absence of molecular testing.

### Study limitations

This study has several limitations. Its retrospective design is associated with missing and heterogeneous data. The small sample size and limited number of events precluded robust multivariate survival analyses. The use of descriptive survival analyses without adjustment introduces a high risk of confounding and indication bias.

## Conclusion

This study highlights major systemic challenges in the management of gastric cancer in Abidjan, including delayed diagnosis, advanced disease at presentation, limited access to multidisciplinary care, absence of molecular testing, and restricted treatment options. Survival outcomes remain poor, reflecting healthcare system constraints rather than treatment efficacy. Strengthening early diagnosis, improving care coordination, and expanding access to diagnostic and therapeutic resources are essential steps toward improving outcomes in resource-limited settings.

## Data Availability

The datasets generated and/or analyzed during the current study are not publicly available due to institutional data protection regulations and patient confidentiality requirements but are available from the corresponding author upon reasonable request.
